# Machine Learning-Based Ultrasomics Improves the Diagnostic Performance in Differentiating Focal Nodular Hyperplasia and Atypical Hepatocellular Carcinoma

**DOI:** 10.3389/fonc.2021.544979

**Published:** 2021-03-26

**Authors:** Wei Li, Xiao-Zhou Lv, Xin Zheng, Si-Min Ruan, Hang-Tong Hu, Li-Da Chen, Yang Huang, Xin Li, Chu-Qing Zhang, Xiao-Yan Xie, Ming Kuang, Ming-De Lu, Bo-Wen Zhuang, Wei Wang

**Affiliations:** ^1^ Department of Medical Ultrasonics, Institute of Diagnostic and Interventional Ultrasound, Ultrasomics Artificial Intelligence X-Lab, The First Affiliated Hospital of Sun Yat-Sen University, Guangzhou, China; ^2^ Department of Traditional Chinese Medicine, The First Affiliated Hospital of Sun Yat-sen University, Guangzhou, China; ^3^ Research Center, GE Healthcare, Shanghai, China; ^4^ Zhongshan School of Medicine, Sun Yat-sen University, Guangzhou, China; ^5^ Department of Hepatobiliary Surgery, The First Affiliated Hospital of Sun Yat-Sen University, Guangzhou, China

**Keywords:** ultrasonography, machine learning, focal nodular hyperplasia, hepatocellular carcinoma, ultrasomics

## Abstract

**Background:**

The typical enhancement patterns of hepatocellular carcinoma (HCC) on contrast-enhanced ultrasound (CEUS) are hyper-enhanced in the arterial phase and washed out during the portal venous and late phases. However, atypical variations make a differential diagnosis both challenging and crucial. We aimed to investigate whether machine learning-based ultrasonic signatures derived from CEUS images could improve the diagnostic performance in differentiating focal nodular hyperplasia (FNH) and atypical hepatocellular carcinoma (aHCC).

**Patients and Methods:**

A total of 226 focal liver lesions, including 107 aHCC and 119 FNH lesions, examined by CEUS were reviewed retrospectively. For machine learning-based ultrasomics, 3,132 features were extracted from the images of the baseline, arterial, and portal phases. An ultrasomics signature was generated by a machine learning model. The predictive model was constructed using the support vector machine method trained with the following groups: ultrasomics features, radiologist’s score, and combination of ultrasomics features and radiologist’s score. The diagnostic performance was explored using the area under the receiver operating characteristic curve (AUC).

**Results:**

A total of 14 ultrasomics features were chosen to build an ultrasomics model, and they presented good performance in differentiating FNH and aHCC with an AUC of 0.86 (95% confidence interval [CI]: 0.80, 0.89), a sensitivity of 76.6% (95% CI: 67.5%, 84.3%), and a specificity of 80.5% (95% CI: 70.6%, 85.9%). The model trained with a combination of ultrasomics features and the radiologist’s score achieved a significantly higher AUC (0.93, 95% CI: 0.89, 0.96) than that trained with the radiologist’s score (AUC: 0.84, 95% CI: 0.79, 0.89, *P* < 0.001). For the sub-group of HCC with normal AFP value, the model trained with a combination of ultrasomics features, and the radiologist’s score remain achieved the highest AUC of 0.92 (95% CI: 0.87, 0.96) compared to that with the ultrasomics features (AUC: 0.86, 95% CI: 0.74, 0.89, *P* < 0.001) and radiologist’s score (AUC: 0.86, 95% CI: 0.79, 0.91, *P* < 0.001).

**Conclusions:**

Machine learning-based ultrasomics performs as well as the staff radiologist in predicting the differential diagnosis of FNH and aHCC. Incorporating an ultrasomics signature into the radiologist’s score improves the diagnostic performance in differentiating FNH and aHCC.

## Introduction

The typical enhancement pattern of hepatocellular carcinoma (HCC) on contrast-enhanced ultrasound (CEUS) is characterized by hyper-enhancement in the arterial phase and wash out during the portal venous and late phases (1). However, atypical variations occur, especially in some well-differentiated tumors, accounting for 5–41% of HCC cases; such lesions may show sustained hyper-/iso-enhancement in the portal venous and late phases and are defined as atypical HCC (aHCC) (2–4). Meanwhile, most benign focal liver lesions show complete hyper- or iso-enhancement in the portal venous and late phases, making differential diagnosis both crucial and challenging (5, 6). This diagnostic difficulty could be resolved using CEUS techniques, such as micro-flow imaging to further characterize the enhancement features in the arterial phase, e.g., a spoke-wheel artery for focal nodular hyperplasia (FNH) and chaotic vessel for HCC (7–10). However, the interpretation of features involves the experience of radiologists, making inter-reader variability inevitable.

In contrast to the traditional practice of treating medical images as pictures intended solely for visual interpretation, radiomics features could reflect not only the macroscopic manifestation but also the cellular and molecular nature of tissues (11–13). Radiomics offers a vast scale of imaging biomarkers that could potentially assist in detecting and diagnosing, evaluating the prognosis and predicting the therapeutic response, and monitoring the disease status of cancer (11, 12, 14–16). Machine learning-based ultrasomics approaches, derived from radiomics, involve the analysis and transformation of ultrasound images into large sets of quantitative data and have been identified as potential alternatives to detect and classify lesions (17, 18).

Recently, few applications of machine learning in HCC diagnosis have been reported (19, 20). Most machine learning systems have demonstrated excellent diagnostic performance, with the area under the receiver operating characteristic curve (AUC) of 0.89-0.97 for HCC characterization (19, 20). Gatos et al. applied radiomics to segment and classify focal liver lesions on non-enhanced T2-weighted images, providing a non-invasive method for assessing liver lesions (21). Some studies have shown that multi-modal ultrasound images also perform well for the detection and classification of focal liver lesions (19, 22, 23). However, most studies have only compared the diagnostic performance between machine learning systems and radiologists. The influence of the performance of these systems on radiologists when used in clinical practice has not been evaluated. Thus, the added clinical value of machine learning systems to observers is necessary to determine and validate.

The purpose of our research was to develop a machine learning-based ultrasomics approach to assess ultrasomics features for improving the diagnostic performance in differentiating FNH and aHCC.

## Patients and Methods

### Patients

This retrospective analysis obtained ethical approval and waived the informed consent requirement. From December 2013 to January 2018, 119 patients with FNH and 107 patients with aHCC lesions were included in the study based on the inclusion and exclusion criteria. The inclusion criteria were as follows: (a) CEUS was performed; (b) lesions were visually hyper-enhanced during the arterial phase and sustained hyper- or iso-enhanced during the portal venous and late phases; (c) HCC was diagnosed by pathological examinations and FNH was confirmed by pathological examinations or supported by CT or MRI findings with a minimum 1 year follow-up; and (d) no treatment was conducted before CEUS. Patients were excluded if they had multiple tumors. Baseline clinical trial data, including age, gender, and some blood test, such as hepatitis background and alpha-fetoprotein (AFP), were performed no more than 7 days before or after the CEUS examination.

### Image Acquisition

US examinations were performed using an Aplio 500 scanner (Canon Medical Systems, Tokyo, Japan), equipped with a 375BT convex transducer (frequency, 3.5 MHz) and an Aixplorer scanner (Supersonic, Paris, France) with an SC6-1 curvilinear transducer (frequency, 1–6 MHz). Contrast harmonic imaging (CHI) and contrast pulse sequencing (CPS) were used with a mechanical index of 0.06–0.10. Baseline ultrasonography was performed to scan the liver thoroughly before CEUS. Additionally, the target lesions were identified and observed carefully during the baseline observation in B mode. The imaging settings, such as the gain, depth, and focus, were optimized for each examination. After the CHI or CPS mode was activated, a bolus intravenous injection of 2.4 mL of SonoVue (Bracco, Milan, Italy) was administered, followed by flushing with 5 mL of saline. The targeted lesion was observed continuously for 5 minutes. The arterial, portal venous, and late phases were defined as 10–30 seconds, 31–120 seconds, and 121–300 seconds after injection, respectively. CEUS examinations were performed by one of two radiologists (WW and X-YX) with at least 10 years’ experience of performing CEUS. Three images from the same section, which showed the maximum observation of the target lesion, were taken from each patient, (a) a baseline ultrasound; (b) an arterial phase image at the enhancement time of 25–30 seconds; and (c) a portal phase image at the enhancement time of 60–70 seconds.

### Radiologist’s Scoring

**Figure 1:**
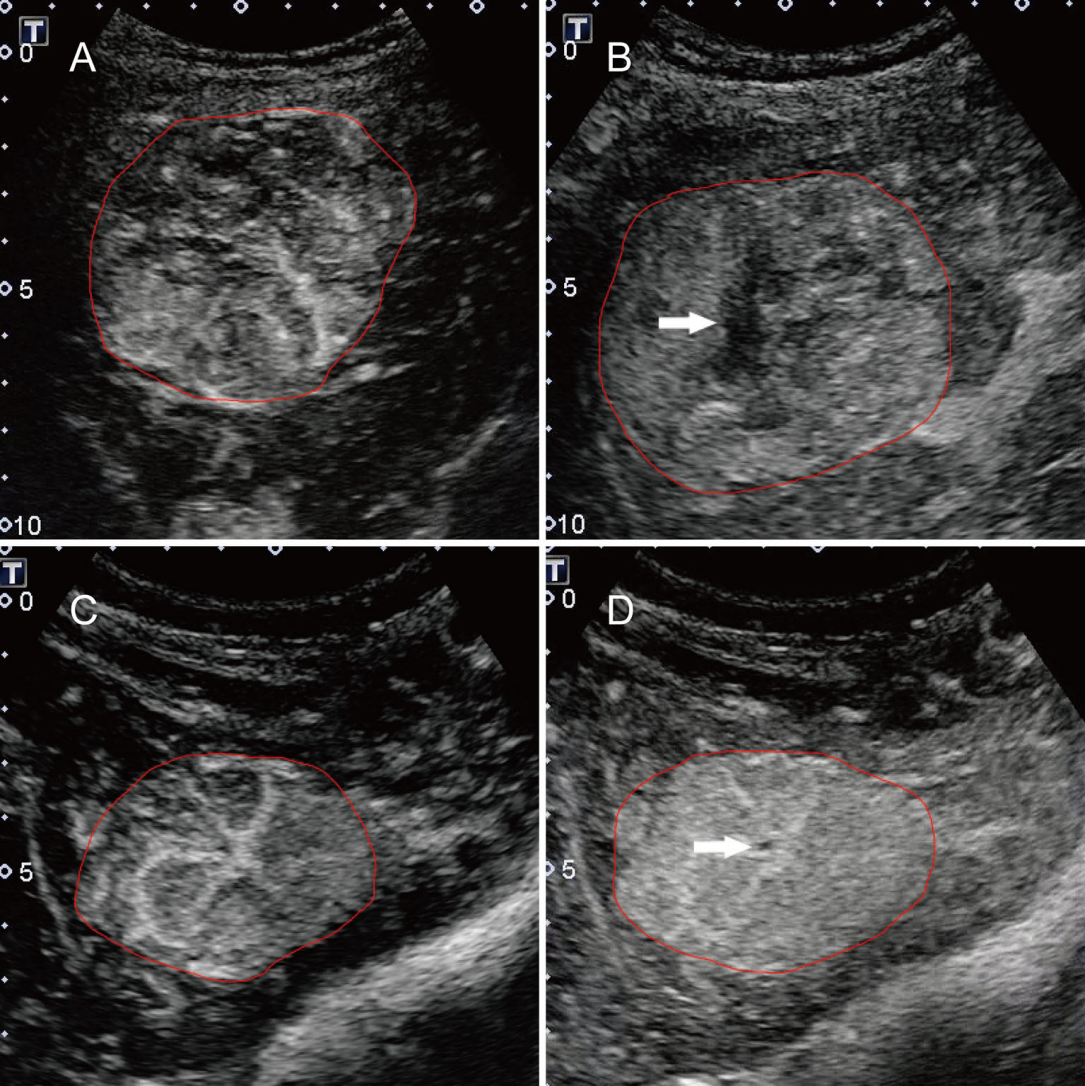
Typical features for HCC and FNH lesions. **(A)** the basket pattern and/or chaotic vessels; **(B)** non-enhancing areas (arrow); **(C)** spoke-wheel arteries; and **(D)** unenhanced central scar (arrow). Annotations of the ROI generated by the radiologists around the tumor outline are delineated in red.

Two staff radiologists (B-WZ and L-DC) reviewed ultrasound images and videos retrospectively, and they had more than 5 years of experience in assessing liver CEUS data. The radiologists were not involved in the feature extraction process below. All patient identification information from the images was removed, and the researchers were unaware of all the clinicopathological information. The diagnostic criteria for HCC and FNH were based on the 2012 guidelines issued by the European Federation of Societies for Ultrasound in Medicine and Biology (EFSUMB) (2). The diagnostic criteria for HCC were the manifestation of basket pattern and/or chaotic vessels (**Figure 1A**) and non-enhanced areas (**Figure 1B**). The diagnostic criteria of CEUS feature for FNH were centrifugal enhancement (Video 1), spoke-wheel artery (**Figure 1C**), unenhanced central scar (**Figure 1D**), and feeding artery.

For each group, the diagnostic confidence was scored using a subjective three-point scale (grade 1, definitely or most likely FNH; grade 2, indeterminate; and grade 3, most likely or definitely HCC). If there was inconsistency, we performed a consensus reading, and the consensus data were used for subsequent analysis.

### Ultrasomics Feature Extraction

Digital imaging and communications in medicine (DICOM) images were used to extract ultrasomics features using the in-house designed Ultrasomics-Platform software (Version 1.0; Ultrasomics Artificial Intelligence X-lab, Guangzhou, China). After an image was imported, the radiologist drew a region of interest (ROI) on the largest cross-section along the tumor contour. Next, the software automatically extracted the features from the ROIs. In total, 1,044 features could be extracted from a single image. These 1,044 features extracted from a single image consisted of five categories of features: histogram parameters, textural parameters, form factor parameters, grey-level co-occurrence matrix (GLCM) parameters, and run length matrix (RLM) parameters. Detailed information on the features is provided in **Supplementary Material S1**. Finally, 3,132 features were extracted from the baseline US, arterial phase and portal phase of CEUS images of each patient. Initially, two radiologists (WL and YH, with at least 5 years of experience in performing US examinations) were required to trace out ROIs on the selected images. The inter-observer and intra-observer reproducibility in feature extraction were assessed and are described in **Supplementary Material S2**. The remaining images were delineated by the first radiologist.

### Feature Selection and Model Development for Prediction

Of the 3,132 features from each patient, many were highly redundant, which could degrade the classification. We eliminated redundant features by using a two-step feature selection method. First, if two features were highly-correlated with a correlation coefficient higher than 0.95, one of the features was removed. Second, we eliminated features with an AUC less than 0.6. According to the Harrell guidelines for multivariate analysis, the number of events should be at least 10 times greater than that of the included covariates (24). The least absolute shrinkage and selection operator (LASSO) regression was used to perform the ultrasomics features selection in the training dataset. All ultrasomics feature values were normalized by using the mean and variance of the feature values to be within similar dynamic ranges.

A support vector machine (SVM) based on the radial basis function (RBF) kernel was trained from the selected feature subset produced by the preceding steps. The entire data set was randomly divided into a training dataset (comprising 80% of subjects) and a validation dataset (comprising the remaining 20% of subjects). The training dataset was used to construct a model, which was then evaluated using the validation dataset. A 10-fold cross-validation method was adopted to ensure the robustness of the classifiers to training and testing data. All processes were repeated 10 times with random seeds, generating 10 different training and validation datasets. We built the model using the training dataset and then evaluated it using the validation dataset repeatedly. Subsequently, the model with the best classification performance was selected as the best model.

### Statistical Analysis

Descriptive statistics are summarized as the mean ± standard deviation (SD) or median and interquartile range. Comparisons between groups were tested using Student’s t test or the Mann-Whitney test for quantitative variables and the chi-squared test or Fisher’s test for qualitative variables.

A weighted kappa statistics test was used to assess the two radiologists’ scores. We evaluated the reproducibility of the ultrasomics feature extraction using the “irr” package in R. The LASSO regression was performed using the “glmnet” package.

All observations of patients with known outcomes were classified into three datasets (1): radiologist’s score, (2) ultrasomics features, and (3) a combination of ultrasomics features and radiologist’s score. The diagnostic performance of the radiologist’s score was evaluated by plotting receiver operating characteristic (ROC) curves. The diagnostic performance in discriminating between FNH and aHCC is expressed as the AUC. The ultrasomics features and the combination of the ultrasomics features and radiologist’s score were further compared through an SVM classifier using the “rattle” package in R. The performance of the SVM model was tested using the AUC. Paired comparisons of AUC values were performed by a two-sided Wilcoxon signed-rank test at a significance level of 5%. The predictive sensitivity (SEN), specificity (SPE), positive predictive value (PPV), negative predictive value (NPV), positive likelihood ratio (+LR), and negative likelihood ratio (-LR) were calculated at a cut-off point that maximized the value of the Youden index. Comparisons among the three datasets were performed using the Delong test. Decision curve analysis (DCA) was performed with the “dca.R” function. All statistical tests were two-sided tests, and *P<* 0.05 indicated statistical significance. All Statistical analyses were performed using R version 3.3.3 (http://www.r-project.org/).

## Results

### Clinical Characteristics

The clinical characteristics are listed in **Table 1**. The study included 226 patients; 107 (47.3%) patients (mean age, 54.0 ± 11.9 years old) had a final diagnosis of HCC; and the remaining 119 (52.7%) patients (mean age, 34.5 ± 11.7 years old) had a final diagnosis of FNH. 20 FNH lesion were confirmed by pathological examinations (11 by biopsy, 9 by surgery), while 99 cases were supported by CT or MRI findings with a minimum one-year follow-up. No significant difference was found in the tumor number between the two groups (*P*=0.118). The average lesion size of FNH and HCC was 3.3 ± 1.8 cm (range: 0.8-10.2 cm) and 4.8 ± 3.4 cm (range: 0.8-18.6 cm), respectively.

**Table 1:** Clinical Characteristics and Laboratory Information of the Patients.

Patients	FNH(N=119)	aHCC(N =107)	P value
**Gender (male/female)**	61/58	92/15	<0.001
**Age (years)**	34.5 ± 11.7	54.0 ± 11.9	<0.001
**HBsAg (IU/ml)**			<0.001
**≤0.05**	116 (97.5)	15 (14.0)	
** 0.05–250**	1 (0.8)	34 (31.8)	
**>250**	2 (1.7)	58 (54.2)	
**HBV-DNA (IU/mL)**			<0.001
**<100**	118 (99.2)	46 (43.0)	
**100–10^5^**	0	44 (41.1)	
**>10^5^**	1 (0.8)	17 (15.9)	
**HCV-Ab (S/CO)**			0.212
**<1.0**	119 (100)	105 (98.1)	
**≥1.0**	0	2 (1.9)	
**AFP (μg/L)**			<0.001
**<20**	117 (98.3)	38 (35.5)	
**20–400**	2 (1.7)	40 (37.4)	
**>400**	0	29 (27.1)	
**Tumor number**			0.118
**1**	112 (94.1)	92 (86.0)	
**2**	4 (3.4)	8 (7.5)	
**≥3**	3 (2.5)	7 (6.5)	
**Tumor size (cm)**	3.34 ± 1.80	4.76 ± 3.35	<0.01
**<3**	64 (53.8)	37 (34.6)	
**3-5**	35 (29.4)	35 (32.7)	
**>5**	20 (16.8)	35 (32.7)	

Data are the number of patients, with the percentage in parentheses unless indicated. aHCC, atypical hepatocellular carcinoma.

### Ultrasomics Signature Construction and Validation

**Figure 2:**
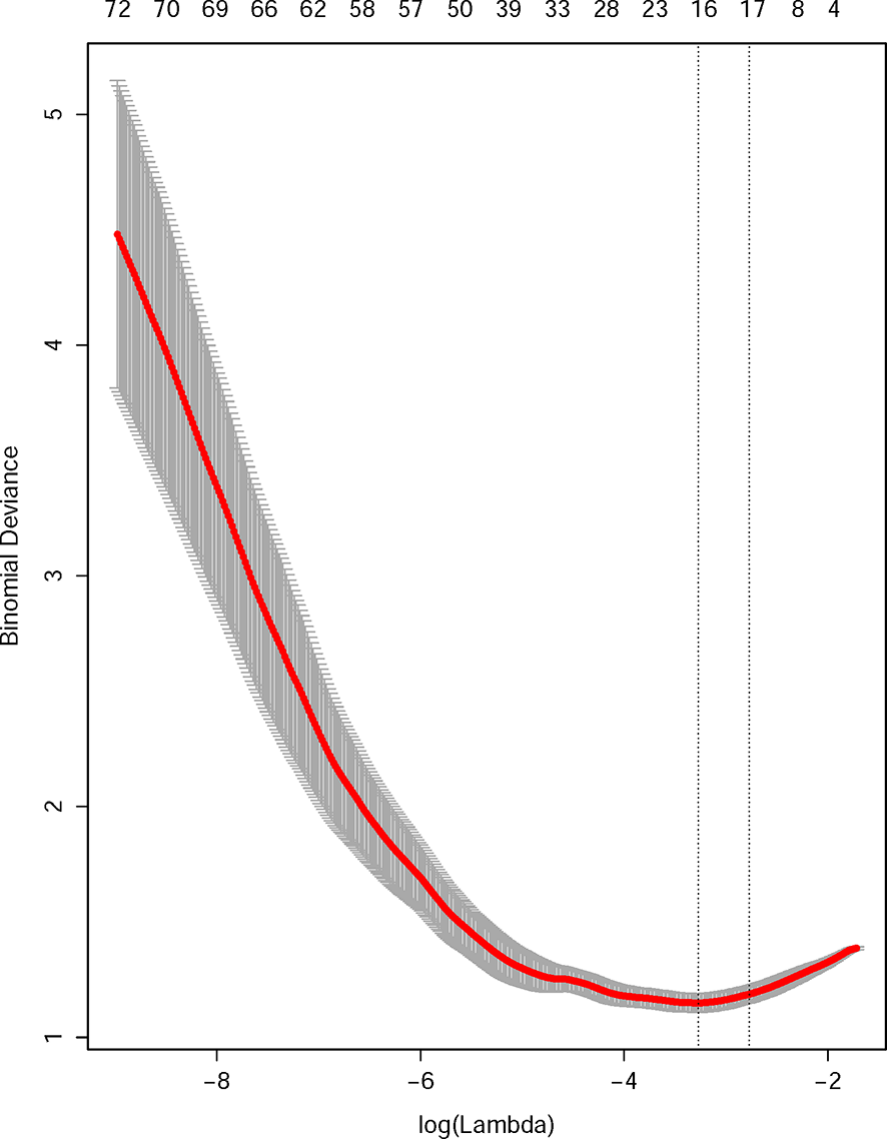
Radiomics feature selection using the least absolute shrinkage and selection operator (LASSO) regression model. The 10-fold cross-validation (CV) process was repeated 50 times to generate the optimal penalization coefficient lambda (λ) in the LASSO model. The value of λ that produced the minimum average binomial deviance was used to select features. Dotted vertical lines were drawn at the optimal values using the minimum criteria and the 1 standard error of the minimum criteria (the 1-SE criteria). A λ value of 0.043 was chosen (the 1-SE criteria) according to 10-fold CV, where optimal λ resulted in 14 nonzero coefficients.

After the feature selection and dimensional reduction process, 14 selected features were taken as the input of the SVM to train a prediction model, including 6 features derived from baseline US images, 3 from arterial phase images, and 4 from portal phase images (**Figure 2**, **Supplementary Material S3**). All feature values were normalized to achieve similar dynamic ranges. The parameter C which is used to control the error-margin trade-off was set at 1, and the kernel width sigma was 0.012. Next, the training and validation procedures for tumor classification were employed with 10-fold cross-validation.

### Diagnostic Performance of Ultrasomics Features and Radiologist’s Score Models

A total of 83 FNHs were correctly identified while 36 lesions were incorrectly identified as HCC, leading to a specificity of 69.8% by the radiologists. For combined model, 100 FNHs were correctly classified whereas 19 lesions were incorrectly assigned to HCC, resulting in a specificity of 84.0%. Comparing the performance of the radiologists' score and the combined model, twenty-four cases have a different result; consequently, the combined model leads to an additional 15 FNHs and 5 HCCs being correctly classified.

**Figure 3:**
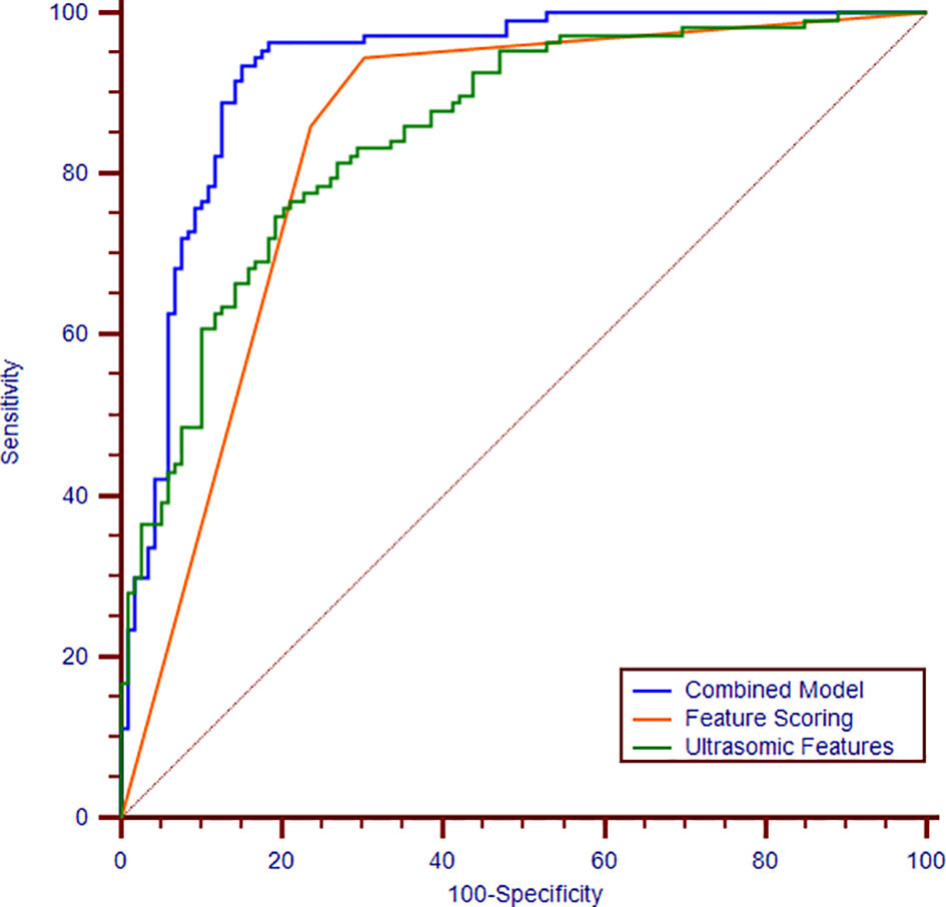
Receiver operating characteristic curves of the combination of ultrasomics features and radiologist’s score (blue curve), ultrasomics features (green curve), and radiologist’s score (orange curve). The areas under the curves are 0.93, 0.86, 0.84, respectively.

The model trained with the combination of the ultrasomics features and radiologist’s score performed significantly better (AUC: 0.93, 95% CI: 0.89, 0.96) than that trained with the ultrasomics features (AUC: 0.86, 95% CI: 0.80, 0.89, *P* < 0.001) and radiologist’s score (AUC: 0.84, 95% CI: 0.79, 0.89, *P* < 0.001). Overall, the model based on the radiologist’s score had the highest diagnostic SEN of 94.4% (95% CI: 88.2%, 97.9%) but the lowest SPE of 69.8% (95% CI: 60.7%, 77.8%) compared with the combined model (SEN: 93.5% [95% CI: 87.0%, 97.3%], SPE: 84.9% [95% CI: 77.1%, 90.8%]), and the ultrasomics features model (SEN: 76.6% [95% CI: 67.5%, 84.3%], SPE: 80.5% [95% CI: 70.6%, 85.9%]). Furthermore, when the ultrasomics features were combined with the radiologist’s score, the diagnostic performance was significantly improved in terms of the AUC, SPE, and PPV and + LR (AUC: 0.93, SPE: 84.9%, and PPV: 84.7%, +LR 6.2) compared with the performance of the other two models. The performance measurements of each dataset are reported in **Table 2** based on each ROC curves to distinguish between FNH and aHCC (**Figure 3**).

**Table 2:** Diagnostic Performance of the Three Models in Differentiating Focal Nodular Hyperplasia and Atypical Hepatocellular Carcinoma.

	Ultrasomics score	Radiologist’s score	Combined
**Sensitivity (%)**	76.6 (67.5-84.3)	94.4 (88.2-97.9)	93.5 (87.0-97.3)
**Specificity (%)**	80.5 (70.6-85.9)	69.8 (60.7-77.8)	84.9 (77.1-90.8)
**PPV (%)**	76.6 (67.5-84.3)	73.7 (65.5-80.9)	84.7 (77.0-90.7)
**NPV (%)**	79.0 (70.6-85.9)	93.3 (85.9-97.5)	93.5 (87.1-97.3)
**+LR**	3.7 (3.2-4.2)	3.1 (2.7-3.5)	6.2 (5.6-6.8)
**-LR**	0.3 (0.2-0.5)	0.1 (0.04-0.2)	0.1 (0.03-0.2)
**AUC of training set**	0.94 (0.89-0.99)	0.93 (0.85-0.98)	0.99 (0.94-1.00)
**AUC of validation set**	0.86 (0.80-0.89)	0.84 (0.79-0.89)	0.93 (0.89-0.96)

Data in parentheses are 95% confidence interval. PPV, positive predictive value; NPV, negative predictive value; +LR, positive likelihood ratio; -LR, negative likelihood ratio; AUC, area under the curve.

**Figure 4:**
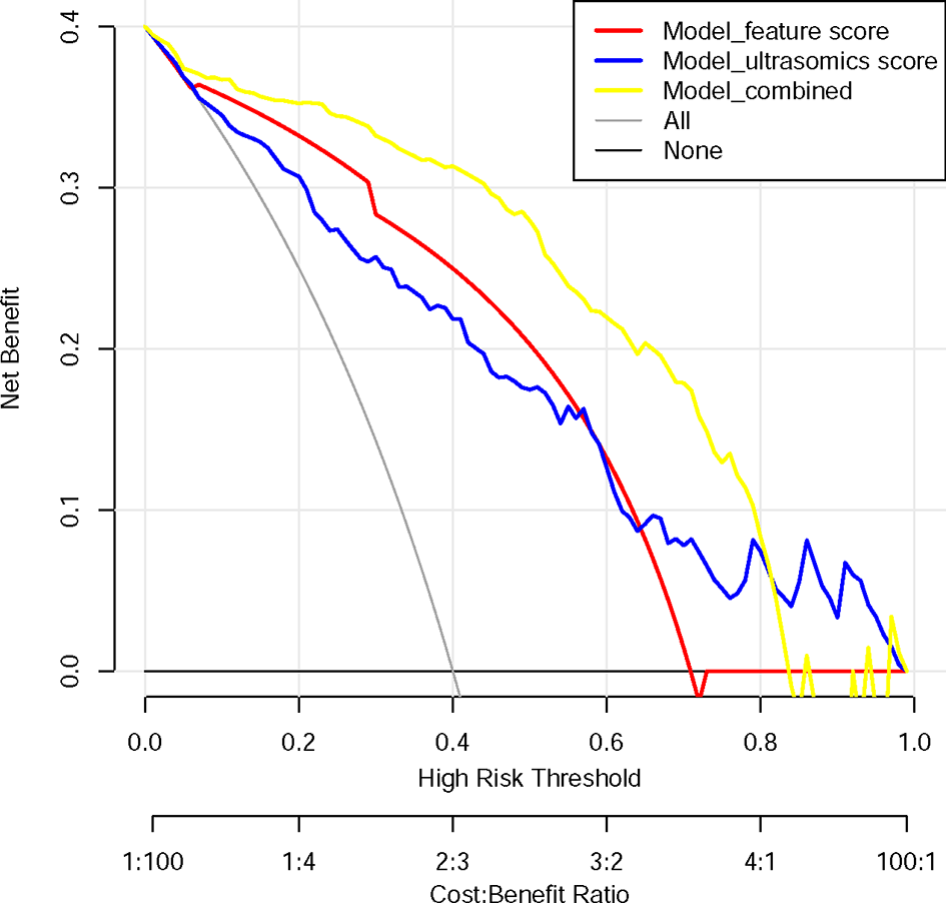
Decision curve analysis for each model. The y-axis measures the net benefit. The net benefit was calculated by summing the benefits (true positive results) and subtracting the harms (false-positive results), weighting the latter by a factor related to the relative harm of undetected cancer compared with the harm of unnecessary treatment. The combined model (yellow line) had the highest net benefit compared with the other two models (blue line and red line) and simple strategies, such as the follow-up of all patients (grey line) or no patients (horizontal black line), across the full range of threshold probabilities at which a patient would choose to undergo a follow-up imaging examination.

The DCA shows that within most reasonable threshold probability ranges, the combined model showed the highest overall net benefit than the radiologist’s score or ultrasound feature model. The DCA results for the three models are presented in **Figure 4**.

### Validation in the Sub-group of HCC With Normal AFP Level

Forty patients were confirmed to develop HCC with normal AFP level. The model trained above performed significantly better (AUC: 0.92, 95% CI: 0.87, 0.96) with the combination of the ultrasomics features and radiologist’s score than that with the ultrasomics features (AUC: 0.86, 95% CI: 0.74, 0.89, *P* < 0.001) and radiologist’s score (AUC: 0.86, 95% CI: 0.79, 0.91, *P* < 0.001) (**Table 3**).

**Table 3:** Validation in the sub-group of HCC with normal AFP value.

	Sensitivity (%)	Specificity (%)	PPV (%)	NPV (%)	+LR	-LR	AUC
**Ultrasomics score**	77.5 (61.5-89.1)	80.5 (72.2-87.2)	60.0 (43.1-75.3)	89.8 (81.5-95.2)	4.4 (3.4-5.8)	0.3 (0.2-0.6)	0.86 (0.74- 0.89)
**Radiologist’s score**	92.5 (79.6-98.3)	77.1 (68.5-84.3)	57.8 (44.7-70.2)	96.8 (90.9-99.3)	4.0 (3.5-4.6)	0.1 (0.03-0.3)	0.86 (0.79-0.91)
**Combined**	95.0 (83.0-99.2)	82.2 (74.1-88.6)	64.4 (50.9 - 76.4)	98.0 (92.9-99.7)	5.3 (4.8-6.0)	0.06 (0.01-0.2)	0.92 (0.87- 0.96)

Data in parentheses are 95% confidence intervals. PPV, positive predictive value; NPV, negative predictive value; +LR, positive likelihood ratio; -LR, negative likelihood ratio; AUC, area under the curve.

## Discussion

In this study, we derived and validated an ultrasomics-based machine learning approach to analyze ultrasound images for the preoperative individualized diagnosis of FNH and aHCC. Our analysis reveals that the diagnostic performance of ultrasomics is comparable to that of a staff radiologist in differentiating between FNH and aHCC. Furthermore, when adding ultrasomics to the radiologist’s classification, the diagnostic performance was improved significantly with an AUC ranging from 0.84 to 0.93 (*P* < 0.001). Our study shows that ultrasomics may increase the diagnostic confidence of radiologists in CEUS examinations and potentially improve their accuracy when facing atypical features. Thus, clinicians would benefit from this decision-making process in the diagnosis of HCC.

In clinical practice, when radiologists face a lesion that shows hyper-enhancement in the arterial phase and sustained enhancement in the portal vein and late phases, it is difficult to diagnose HCC. However, the high sensitivity and negative predictive values of the radiologist would be useful in clinical practice for excluding disease; thus, HCC would be excluded by the radiologist if the result was considered to be FNH by ultrasomics. Therefore, this system would help to reduce unnecessary biopsies or active clinical treatment requested by experienced radiologists.

In contrast, ultrasomics, referred to as high-throughput computing, extracts innumerable quantitative features from US images (18). By transforming digital medical images into mineable high-dimensional data, ultrasomics yields features, such as textural features, that could objectively reflect the homogeneity or heterogeneity of an image. These patterns could represent enhancement features just as heterogeneity might represent chaotic vessels and necrosis. Focal liver lesions can be featured by typical features in the arterial phase and wash-out during the portal and late phases. In this study, the features displayed in the arterial phase could provide a major benefit for the diagnosis of liver tumors. In previous studies, we utilized a maximum intensity projection technique of micro-flow imaging and achieved higher spatial resolution and higher temporal resolution when detecting vessel contours. Compared to conventional CEUS features (AUC: 0.84), micro-flow imaging technology provided significant improvements over the detection rates achieved for the staff radiologists (AUC: 0.89) (10). In this study, ultrasomics features alone can achieve a similar diagnostic performance (AUC: 0.86) as micro-flow imaging (AUC: 0.868–0.873). Ultrasomics could reach such achievement because it analyzes textural features objectively and quantitatively to describe the intrinsic characteristics of tumors, in particular heterogeneous tumors. Ultrasomics analysis has already been applied to various types of disease, such as HCC, liver fibrosis, and breast cancer (18, 25–27). The potential of ultrasomics has already been demonstrated for liver imaging in some studies (17, 18).

In this study, we additionally evaluated the benefit of ultrasomics in assisting doctors with the interpretation of medical images. As an interdisciplinary technology, it combines elements of imaging generation, digital image processing, statistical imaging, and knowledge engineering to manage the volume of information related to the diagnostic process and outcome prediction (17, 18, 25). In our study, the additional information in the combined model led to improved diagnostic performance (AUC: 0.93) and higher specificity of 84.9% compared with the ultrasomics (AUC: 0.86, specificity: 80.5%) and radiologist’s score (AUC: 0.84, specificity: 69.8%) models. The combined model was also comparable to the ML model based on multi-modal ultrasound images (AUC: 0.94, sensitivity: 91.0%, specificity: 86.0%) (19). The results are also comparable to and even better than those of MRI (AUC: 0.89, sensitivity: 82.2%, specificity: 71.4%), as previously reported (20). However, the use of artificial intelligence is not intended to replace expert diagnosticians because no solution is guaranteed and knowledge-based maintenance is required. Artificial intelligence is also affected by several elements, such as the source of images and the cognition of disease. Presently, most domains of large data have not tapped the full potential of artificial intelligence technology. However, rapid developments in the area will add more potential to the advantages. Therefore, the most important role of artificial intelligence is to help improve diagnostic accuracy and assist rather than replace clinicians in making treatment decisions. It is worth noting that the combined model greatly improved the diagnostic ability of radiologists. A similar conclusion was obtained in another study (28).

Our research has some limitations. First, this study was retrospective and conducted in one center. This may cause potential variations and selection bias in the patient population and imaging methods, which is difficult to generalize the outcomes to other agencies. Second, due to the relative rarity of aHCC, the sample size is relatively small, which may cause over-fitting to this particular population. Hence, large-scale multicenter studies are necessary for the future to validate the results. Third, only two radiologists were involved in the assessment of the basic imaging features and feature extraction. All outcomes were based on the features extracted by one radiologist, which may not be generalizable to all radiologists. Fourth, the machine and imaging settings in this study were inconsistent, which may affect the ultrasomics features (29, 30).

In conclusion, an ultrasomics approach was developed to investigate the association between the quantitative ultrasound features and pathological characteristics of tumors effectively and objectively. We evaluated the added value of ultrasomics to the radiologist, and this approach improved the performance of CEUS by providing quantitative and standardized criteria to radiologists, thereby enabling the more confident application of CEUS in detecting HCC to achieve better treatment planning. Our findings can assist clinicians in the differential diagnosis between FNH and aHCC accurately using CEUS images, and this allows for early and precise medical management and treatment.

## Data Availability Statement

The raw data supporting the conclusions of this article will be made available by the authors, without undue reservation.

## Ethics Statement

The studies involving human participants were reviewed and approved by the Institutional Review Board of the First Affiliated Hospital of Sun Yat-Sen University. The patients/participants provided their written informed consent to participate in this study.

## Author Contributions

WW, M-DL, MK, and X-YX contributed conception and design of the study. XZ collected data from clinical trials. WL and B-WZ drafted and revised the manuscript. X-ZL, WW, L-DC, and S-MR revised the manuscript for important intellectual content. X-ZL, XL, H-TH, YH, and C-QZ gave technical support. All authors contributed to the article and approved the submitted version.

## Funding

This study was supported by grants from the National Natural Science Foundation of China (No. 81601500 and 81701701).

## Conflict of Interest

XL was employed by GE Healthcare.

The remaining authors declare that the research was conducted in the absence of any commercial or financial relationships that could be construed as a potential conflict of interest.
