# Supplementary material for: Machine Learning-Based Ultrasomics Improves the Diagnostic Performance in Differentiating Focal Nodular Hyperplasia and Atypical Hepatocellular Carcinoma
Source: Front Oncol. 2021 Mar 26;11:544979. doi: 10.3389/fonc.2021.544979 (PMC8033198; doi:10.3389/fonc.2021.544979)
Supplement: Supplementary file 1 [file DataSheet_1.docx]

**Supplementary Materials**

1. Feature extraction of ultrasomics

1.1 Histogram parameters

1.2 Texture parameters

1.3 Form Factor parameters

1.4 GLCM parameters

1.5 RLM parameters

2. Reproducibility

3. Selected features in the model

**1. Feature extraction of ultrasomics**

DICOM images were used to extract ultrasomics features using the in-house designed Ultrasomics-Platform software (Version 1.0; Ultrasomics Artificial Intelligence X-lab, Guangzhou, China). After importing an image, the radiologist drew a region of interest (ROI) around the tumor outline. Then, the software automatically extracted the features from the ROIs. In total, 1,044 features were finally extracted from one single image. These 1,044 features consisted of five categories of features: histogram parameters, textural parameters, form factor parameters, GLCM parameters, and RLM parameters. Finally, a total of 3132 features which were extracted in baseline US, arterial phase and portal phase respectively for each patient.

**1.1 Histogram parameters**

Histogram parameters describe the distribution of grayscale pixel intensities within the conventional image through commonly used and basic metrics. To analyze the distribution of the pixels’ hue matrix and extract static features of images, a fuzzy similitude matrix is defined. The matrix describes the image’s feature distribution. The following statistics features were extracted: energy, entropy, max intensity, min intensity, mean value, mean absolute deviation, median intensity, range, root mean square (RMS), standard deviation, uniformity, variance, volume count, voxel value sum, relative deviation, frequency size, quantiles, percentiles, skewness, kurtosis.

**1.2 Texture parameters**

Texture is one of the important characteristics used in identifying objects or regions of interest in an image, texture represents the appearance of the surface and how its elements are distributed. Various texture analysis approaches tend to represent views of the examined textures form different perspectives, and due to multi-dimensionality of perceived texture, there is not an individual method that can be sufficient for all textures. The following statistics features were extracted: energy, entropy, correlation, inertia, cluster shade, and cluster prominence.

**1.3 Form factor parameters**

These group of features includes descriptors of the three-dimensional size and shape of the tumor region. We determined the following shape and size-based features: sphericity, surface area, compactness, maximum 3D diameter, spherical disproportion, surface to volume ratio, and volume.

**1.4 GLCM parameters**

The Grey level co-occurrence matrix (GLCM) is the matrix function that describes the distance and angle of each pixel and can reflect integrated information regarding the direction, interval, amplitude, and frequency of images. The advantage of the co-occurrence matrix calculations is that the co-occurring pairs of pixels can be spatially related in various orientations with reference to distance and angular spatial relationships, as on considering the relationship between two pixels at a time. As a result, the combination of grey levels and their positions are exhibited apparently. Therefore, it is defined as “A two-dimensional histogram of gray levels for pair of pixels, which are separated by a fixed spatial relationship”. However, the matrix is sensitive to rotation. With the change of different offsets define pixel relationships by varying directions. The rotation angle of an offset: 0°,45°,90°,135° and displacement vectors, different co-occurrence distributions from the same image of reference. GLCM of an image is computed using displacement vector d defined by its radius, (distance or count to the next adjacent neighbor preferably is equal to one) and rotational angles. The following statistics categories were extracted: GLCM energy, GLCM entropy, inertia, correlation, inverse difference moment, and Haralick features.

**1.5 RLM parameters**

The grey level run-length matrix (RLM) 𝐏_𝐫_(𝐢,𝐣 | 𝛉 ) is defined as the numbers of runs with pixels of gray level i and run length j for a given direction θ. RLMs is generated for each sample image segment having directions (0°,45°,90° &135°), then the following ten statistical features were derived: short run emphasis, long run emphasis, grey level non-uniformity, run length non-uniformity, Low Grey Level Run Emphasis, High Grey Level Run Emphasis, Short Run Low Grey Level Emphasis, Short Run High Grey Level Emphasis, Long Run Low Grey Level Emphasis and Long Run High Grey Level Emphasis.

**2. Reproducibility**

To evaluate inter-observer and intra-observer reproducibility of ultrasomics feature extraction, two radiologists with 10 (reader 1) and 12 years (reader 2) of experience in abdominal US interpretation, independently drew the ROI of focal liver lesions on the images with maximum diameter of the lesion. To evaluate intra-observer reproducibility, reader 1 drew the ROI of focal liver lesions for images twice in a week period. An independent samples t-test or Kruskal-Wallis H test, where appropriate, was used to assess the differences between the features generated by reader 1 (first time) and those by reader 2, as well as between the twice-generated features by reader 1. Inter- and intra-class correlation coefficients (ICCs) were used to evaluate the intra- and inter-observer agreement of features extraction. The ICC was categorized as follows: slight, 0 ≤ ICC < 0.20; fair, 0.20≤ ICC < 0.40; moderate, 0.40 ≤ICC < 0.60; substantial, 0.60≤ICC < 0.80; and almost perfect, ICC ≥0.80.

**2.1 Feature Scoring Agreement**

The overall inter-reader agreement was moderate for radiologist’s scores between the two readers (Kappa = 0.665; 95% CI 0.571–0.678; *P* < 0.001).

**2.2 Ultrasomics Reproducibility**

Satisfactory inter- and intra-observer reproducibility of the ultrasomics features extraction was achieved. No statistically significant difference was found neither between the twice features from the reader 1 nor between the first-batch extracted features of the reader 1 and those of the reader 2 (*P*-values ranged from 0.248 to 0.547). The intra-observer ICC calculated based on reader 1’s twice feature extraction ranged from 0.847 to 0.915. The inter-observer ICCs calculated on the basis of reader 1’s first-batch extracted features and those of the reader 2 ranged from 0.724 to 0.837. Therefore, all outcomes were based on the features extracted by reader 1.

**3. Selected features in the model**

| Phase | Parameters |
| --- | --- |
| Baseline US | Correlation_angle90_offset1 |
|  | GLCMEntropy_angle0_offset2 |
|  | Correlation_AllDirection_offset4_SD |
|  | GLCMEntropy_angle135_offset9 |
|  | SumVariance |
|  | ShortRunHighGreyLevelEmphasis_AllDirection_offset2_SD |
| Arterial phase | Correlation_AllDirection_offset4_SD |
|  | LongRunEmphasis_angle90_offset3 |
|  | LongRunHighGreyLevelEmphasis_AllDirection_offset6_SD |
|  | LongRunEmphasis_angle135_offset7 |
| Venous phase | LongRunHighGreyLevelEmphasis_AllDirection_offset3_SD |
|  | LongRunHighGreyLevelEmphasis_AllDirection_offset4_SD |
|  | LongRunHighGreyLevelEmphasis_AllDirection_offset6_SD |
|  | LongRunHighGreyLevelEmphasis_angle135_offset8 |
